# Phthalates in Indoor Dust and Their Association with Building Characteristics

**DOI:** 10.1289/ehp.7809

**Published:** 2005-06-01

**Authors:** Carl-Gustaf Bornehag, Björn Lundgren, Charles J. Weschler, Torben Sigsgaard, Linda Hagerhed-Engman, Jan Sundell

**Affiliations:** 1 Swedish National Testing and Research Institute, Borås, Sweden; 2 International Centre for Indoor Environment and Technology, Technical University of Denmark, Lyngby, Denmark; 3 Department of Public Health Sciences, Karlstad University, Karlstad, Sweden; 4 Environmental and Occupational Health Sciences Institute, University of Medicine and Dentistry of New Jersey/Robert Wood Johnson Medical School and Rutgers University, Piscataway, New Jersey, USA; 5 Department of Environmental and Occupational Medicine, Aarhus University, Aarhus, Denmark

**Keywords:** BBzP, building characteristics, DEHP, DnBP, homes, PVC flooring, sources

## Abstract

In a recent study of 198 Swedish children with persistent allergic symptoms and 202 controls without such symptoms, we reported associations between the symptoms and the concentrations of *n*-butyl benzyl phthalate (BBzP) and di(2-ethylhexyl) phthalate (DEHP) in dust taken from the childrens’ bedrooms. In the present study we examined associations between the concentrations of different phthalate esters in the dust from these bedrooms and various characteristics of the home. The study focused on BBzP and DEHP because these were the phthalates associated with health complaints. Associations have been examined using parametric and nonparametric tests as well as multiple logistic regression. For both BBzP and DEHP, we found associations between their dust concentrations and the amount of polyvinyl chloride (PVC) used as flooring and wall material in the home. Furthermore, high concentrations of BBzP (above median) were associated with self-reported water leakage in the home, and high concentrations of DEHP were associated with buildings constructed before 1960. Other associations, as well as absence of associations, are reported. Both BBzP and DEHP were found in buildings with neither PVC flooring nor wall covering, consistent with the numerous additional plasticized materials that are anticipated to be present in a typical home. The building characteristics examined in this study cannot serve as complete proxies for these quite varied sources. However, the associations reported here can help identify homes where phthalate concentrations are likely to be elevated and can aid in developing mitigation strategies.

For almost a quarter-century, phthalate esters have been recognized as major indoor pollutants (Clausen et al. 2003; Fromme et al. 2004; Rudel et al. 2003; Wensing et al. 2005; Weschler 1980, 1984). This reflects their widespread use, primarily as plasticizers, in products ranging from polyvinyl chloride (PVC) flooring to vinyl toys. Worldwide phthalate production has been estimated to exceed 3.5 million tons/year (Cadogan and Howick 1996). Different phthalate esters have different chemical and physical properties and, consequently, have different uses. Di(2-ethylhexyl) phthalate (DEHP) accounts for roughly 50% of overall phthalate production, although this percentage has been decreasing in recent years. Most of the current DEHP production is used in PVC products, including PVC flooring, where it typically constitutes 30% of PVC by weight [Cadogan and Howick 1996; Kavlock et al. 2002b; National Toxicology Program (NTP) 2003]. The production of *n*-butyl benzyl phthalate (BBzP) and di-*n*-butyl phthalate (DnBP) is about one-tenth that of DEHP. BBzP is also used as a plasticizer for PVC flooring, as well as for vinyl tile, carpet tiles, and artificial leather and in certain adhesives (Kavlock et al. 2002a). DnBP is used in latex adhesives, as a plasticizer in cellulose plastics, as a solvent for certain dyes, and, to a lesser extent than DEHP, as a plasticizer in PVC (Kavlock et al. 2002c).

Health concerns related to phthalate ester exposures have focused primarily on cancer and reproductive effects (Kavlock et al. 2002a, 2002b, 2002c; NTP 2003). However, phthalate exposures have also been postulated to have a role in the pathogenesis of asthma (Oie et al. 1997), and plasticized indoor materials have been associated with the development of bronchial obstruction in young children (Jaakkola et al. 1999). We recently reported an association between asthma and allergies in children and phthalate concentrations in dust collected from the children’s bedrooms (Bornehag et al. 2004b). The geometric mean concentrations of BBzP were higher in dust from rooms of children with rhinitis compared with controls (0.237 vs. 0.157 mg/g dust, *p* = 0.001) and of children with eczema compared with controls (0.224 vs. 0.157 mg/g dust, *p* = 0.001). Regarding DEHP, dust from rooms of children with asthma had a higher geometric mean concentration compared with that of controls (0.966 vs. 0.741 mg/g dust, *p* = 0.022). For these associations, a dose–response relationship was supported by trend analyses (*p* < 0.05) when the phthalate concentrations in dust were divided into quartiles. DnBP was not associated with doctor-diagnosed disease. Related to these findings, various di- and monophthalate esters have been shown to have an adjuvant effect in a mouse model (Larsen et al. 2001a, 2001b, 2002, 2003), to enhance the production of interleukin-4 in mouse T-cells (Lee et al. 2004), and to potentiate the response of allergic effector cells (Glue et al. 2005).

The aim of the present study was to examine associations between the concentration of phthalates in dust from Swedish homes and selected building characteristics.

## Materials and Methods

### Selection of buildings.

The study is based on 390 homes that participated in the nested case–control study of 400 children in Sweden (Bornehag et al. 2004a). The cases and controls were selected from phase 1 of the Dampness in Buildings and Health (DBH) study. This was a cross-sectional questionnaire study soliciting health and environmental information regarding all 14,077 children 1–6 years of age in the county of Värmland, Sweden; responses were obtained for 10,852 children (Bornehag et al. 2003, 2005a).

The selection criteria for the cases in DBH phase 2 were, in the initial questionnaire, reports of at least two symptoms of “wheezing during last 12 months without a cold,” “rhinitis during last 12 months without a cold,” and “eczema during last 12 months.” In the follow-up questionnaire 1.5 years later, cases had to report at least two of three possible symptoms. Inclusion criteria for the controls were no symptoms in the first questionnaire and no symptoms in the follow-up questionnaire. Both cases and controls must not have rebuilt their homes because of moisture problems, and not have changed residence since the first questionnaire. This process ultimately yielded 198 cases and 202 controls, living in 390 homes.

Factors associated with participation in the study included a greater number of health problems in the case families. Furthermore, in both case and control families, participation was associated with more health-conscious lifestyle factors such as nonsmoking parents and cotton diapers for the child. Higher socioeconomic status, as a selection factor, was indicated by a higher participation among families living in single-family houses compared with multifamily houses, and higher participation among families with two parents living in the home compared with single parent homes (Bornehag et al., unpublished data).

### Building investigations.

There were 10 pairs of siblings among the 400 children; hence, they lived in 390 buildings. Between October 2001 and April 2002, six professional inspectors performed visual inspections and indoor air quality assessments, including dust sampling, in the homes. The inspectors were blinded to case–control status of the children living in the homes. During these investigations, a checklist was followed regarding factors such as the type of building, building construction, building materials, type of ventilation, and mold and moisture problems.

For each residence, 1-week average ventilation rates of both the whole home and the bedroom of the index child were measured using a passive tracer gas method (Nordtest 1997).

### Phthalates in dust.

Samples of dust from 390 homes were collected from moldings and shelves in the children’s bedroom. All dust was sampled during heating season from October 2001 to April 2002. The dust was collected on 90-mm membrane filters made of pure cellulose in holders made of styrene-acrylonitrile polymer mounted on a sampler made of polypropylene (VacuuMark disposable nozzle; Petersen Bach, Bjerringbro, Denmark) connected to a vacuum cleaner. The filters were first packed in aluminum foil and then in a polyethylene bag and stored in a refrigerator for 2–3 days. The filter was weighed before and after sampling under controlled conditions. Before weighing, the filter samples were conditioned at 23°C and 50% relative humidity.

From the 390 homes there were 9 missing samples, 13 samples with errors in the laboratory analysis, and 6 samples with a negative dust weight. Consequently, there were 362 valid samples. Only filters with a net increase in weight of ≥ 25 mg were included in the present analysis; 346 of the 362 dust samples met this criterion.

The dust samples were extracted in pre-cleaned 10-mL glass vials for 30 min using 2 mL dichloromethane. This procedure was repeated, and the two extracts were then combined and transferred to 3-mL autosampler vials. Aliquots from these vials were injected into either a gas chromatograph/mass selective detector for phthalate identification or a gas chromatograph/flame ionization detector for quantitation. The dust concentrations (mg/g dust) were determined for six phthalates: diethyl phthalate (DEP), diisobutyl phthalate (DIBP), DnBP, BBzP, DEHP, and diisononyl phthalate (DINP). For further details regarding chemical analyses, see Bornehag et al. (2004b).

### Statistical method.

We performed analyses of potential associations between concentrations of phthalates in dust and building characteristics using nonparametric tests (Mann-Whitney *U*-test). Log-transformed, normally distributed concentrations (where concentrations below the detection limit have been excluded) were tested with parametric tests (*t*-test) and Pearson correlation (*r*). The analyses were considered statistically significant when *p* < 0.05. The concentrations are reported as medians, as arithmetic means, and as geometric means with 95% confidence intervals (CIs). The CIs were calculated with a back-transform of mean log ± 2 × SE.

We used multiple logistic regression (backward elimination) for analyzing associations between a high phthalate concentration in dust (above median concentration) and building characteristics: PVC as flooring material in the child’s bedroom (no, yes), type of building (single-family house, multifamily houses), construction period (before 1960before 1960–1983, after 1983), and ventilation rate (in quartiles). Data on water leakage in the home during the previous 3 years was collected in the DBH phase 1 questionnaire, 18–24 months before the exposure measurements were conducted.

The study was approved by the ethics committee in Orebro, Sweden.

## Results

Descriptions of the 390 homes included in this case–control study are presented in Table 1. The buildings were primarily single-family houses, and almost 50% of these buildings were constructed before 1960. PVC flooring was the most commonly used flooring material, followed by wood flooring and laminate. There was little difference in the frequency of PVC use between single-family houses and chain houses, but PVC was more commonly used in multifamily houses compared with either of these.

Table 2 lists the phthalate concentrations in dust collected from 346 children’s bedrooms; these were the dust samples that met the criteria for reliable analyses (see “Materials and Methods”). The most frequently identified phthalate was DEHP, which was found in nearly all samples; DnBP was found in 89%, and BBzP was found in 79% of the samples. DEHP also had the highest average concentration in the dust, with a median concentration of 0.77 mg/g dust. All other phthalates were detected at median concentrations below 0.2 mg/g dust. DnBP, BBzP, and DEHP were not highly correlated with each other (r 2 < 0.35).

### Surface materials.

The distribution of surface materials on floors and walls in the bedrooms of cases and controls are presented in Table 3. Significantly more PVC and less wood flooring were found among the cases. This difference is due partly to selection bias. However, the earlier reported association between phthalates in dust and asthma/allergic symptoms among children is not a consequence of either selection bias or active avoidance of specific flooring materials because of allergic disease in the family (Bornehag et al. 2005c). In the instance of vinyl as a wall material, no difference was found between cases and controls, and no selection bias was found. Additionally, more painted wallpaper and less painted glass fiber wallpaper were found among the cases.

**Figure 1:**
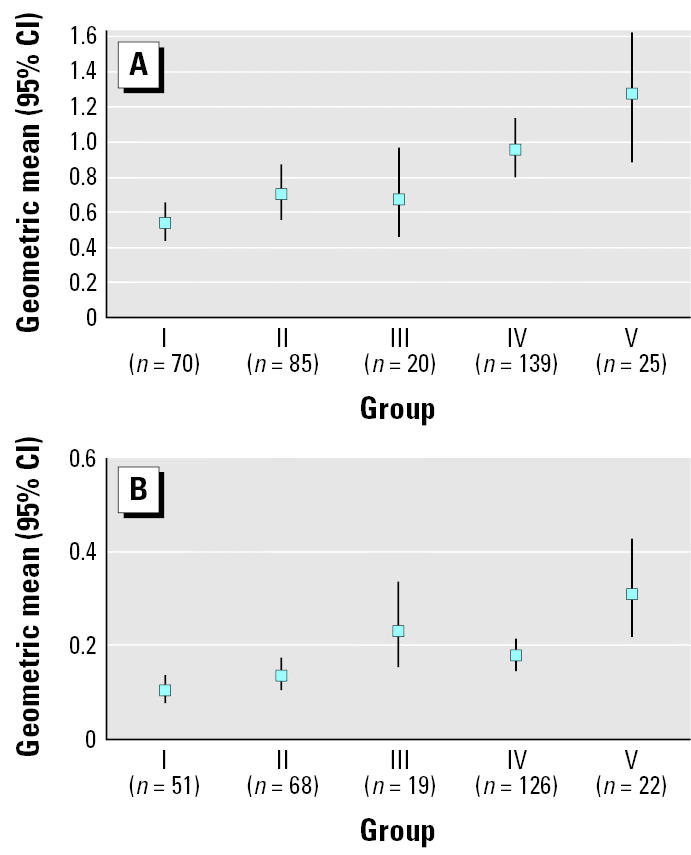
Geometric mean concentration (95% CI) of (A) DEHP and (B) BBzP in surface dust (mg/g dust) in homes with different combinations of flooring material.

As shown in the last three columns of Table 2, the median concentrations of BBzP and DEHP in dust were significantly higher in bedrooms with PVC flooring compared with other flooring materials. In the case of the other four identified phthalates, there were no significant differences between bedrooms with and without PVC flooring. The more rooms with PVC flooring in the home, the higher the geometric mean dust concentrations of both DEHP and BBzP. The particular characteristics of the groups are as follows: group I, no PVC in the child’s bedroom and no PVC in other rooms (parent’s bedroom, living room, kitchen, and hall); II, no PVC in child’s bedroom and PVC in at least one of the other rooms; III, PVC in the child’s bedroom and no PVC in other rooms; IV, PVC in the child’s bedroom and PVC in at least one of the other rooms; V, PVC in the child’s bedroom and PVC in all other rooms. This is illustrated in Figure 1. The association between PVC flooring and the concentration of phthalates in the dust was stronger for BBzP than for DEHP.

The data in Table 2 and Figure 1 also illustrate that PVC flooring is not the only source of BBzP and DEHP in the dust. When there is no PVC flooring in the bedroom, the median amount of DEHP in the dust is 0.7 mg/g; when there is no PVC flooring anywhere in the house, the median amount of DEHP in the dust is 0.55 mg/g. Hence, there is a large background concentration of DEHP to which the DEHP from PVC flooring is contributing. The background concentration for BBzP is not as large. When there is no PVC flooring in the bedroom, its dust concentration is 0.089 mg/g; when there is no PVC flooring anywhere, its dust concentration is comparable (0.100 mg/g).

**Figure 2:**
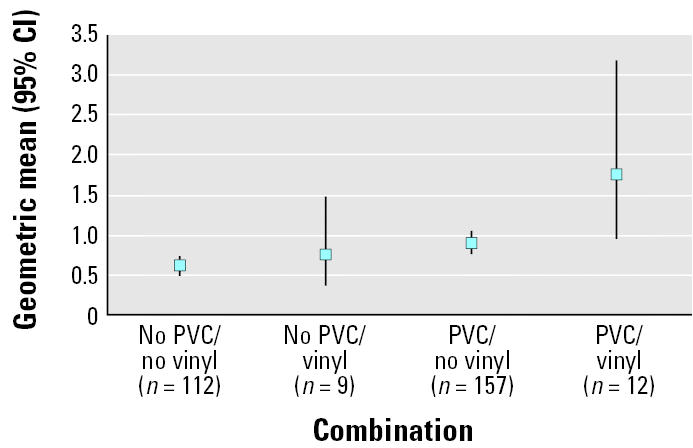
Geometric mean concentration (95% CI) of DEHP in surface dust (mg/g dust) in homes with different combinations of flooring (PVC vs. no PVC) and wall materials (vinyl vs. no vinyl).

Of the 26 homes with vinyl on walls in the child’s bedroom, 12 had PVC as flooring material in the same room. Homes with vinyl on the wall in the child’s bedroom had a higher concentration of DEHP in the dust compared with bedrooms that had other types of wall coverings [1.24 mg/g dust (n = 26; 95% CI, 0.79–1.96) vs. 0.74 mg/g dust (n = 319; 95% CI, 0.67–0.83), p = 0.009 by t-test]. There was no significant difference with wall coverings for BBzP. The highest concentration of DEHP was found in bedrooms with a combination of PVC on the floor and vinyl on the walls (Figure 2).

### Type of building and construction period.

The concentrations of DnBP, BBzP, and DEHP were higher in multifamily houses than in single-family houses, but the differences did not reach significance. Neither were there any significant differences in phthalate concentrations between buildings from different construction periods (i.e., before 1960i.e., before 1961–1983, and after 1983). However, when including only homes with PVC as flooring material in the child’s bedroom, the geometric mean concentrations of DEHP and BBzP were significantly higher in buildings erected before 1960 [DEHP: 1.25 mg/g dust (n = 72; 95% CI, 0.97–1.61); BBzP: 0.25 mg/g dust (n = 60; 95% CI, 0.19–0.33)] compared with buildings constructed after 1983 [DEHP: 0.79 mg/g dust (n = 32; 95% CI, 0.61–1.03); BBzP: 0.15 mg/g dust (n = 32; 95% CI, 0.11–0.20); both p < 0.05 by t-test].

### Type of foundation.

Different types of foundation may produce different moisture loads in a building. Moisture from the ground and/or construction materials such as concrete may have an impact on PVC flooring via various degradation processes (e.g., hydrolysis of phthalate plasticizers). Data on the type of foundation were available only for single-family houses. In such buildings, a significantly higher geometric mean dust concentration of BBzP was found in buildings with a concrete slab on the ground as the foundation [0.20 mg/g dust (n = 72; 95% CI, 0.16–0.26)] compared with buildings with a basement [0.13 mg/g dust (n = 90; 95% CI, 0.11–0.16); p < 0.01 by t-test]. Furthermore, buildings with a concrete slab on the ground had a higher geometric mean concentration of BBzP compared with those with a crawl space; however, the difference did not reach significance (p = 0.077 by t-test).

### Ventilation.

There was no association between the geometric mean concentration of BBzP and the mean ventilation rate (during a week) in the child’s bedroom, but the geometric mean concentration of DEHP was higher in buildings with higher ventilation rates. No association was found between the type of ventilation system and the concentration of phthalates in dust. There was no association between phthalate concentrations in dust and the relative humidity or the temperature in the child’s bedroom.

### Self-reported water leakage.

Homes with self-reported water leakage during the preceding 3 years had higher geometric mean concentrations of BBzP and DEHP in dust than did buildings without such reports [BBzP: 0.19 mg/g dust (n = 67; 95% CI, 0.16–0.24) vs. 0.15 mg/g dust (n = 202; 95% CI, 0.13–0.17), p = 0.049 by t-test; DEHP: 0.93 mg/g dust (n = 78; 95% CI, 0.77–1.13) vs. 0.75 mg/g dust (n = 242; 95% CI, 0.66–0.85), p = 0.084 by t-test]. When the analysis included buildings with only PVC as the flooring material in the child’s bedroom, the association became somewhat stronger (by t-test: BBzP, p = 0.012; DEHP, p = 0.062).

### Multivariate analyses.

Table 4 displays associations between building characteristics and the dust concentrations of BBzP or DEHP as determined by multiple logistic regression models. (Data on type of foundation were not included in the models because such data were available only for single-family houses.) In these analyses the dependent variable (i.e., the concentration of phthalate in the dust) was divided into two groups: low, below the median concentration, and high, above the median concentration. In a backward stepwise logistic regression, high BBzP concentration was associated with PVC flooring and, to a lesser degree, with self-reported water leakage during the previous 3 years. Elevated DEHP concentration was associated with PVC flooring and with home construction before 1960. In the univariate multiple logistic regression, ventilation rate was associated with DEHP in dust. However, in the adjusted model such an association disappeared. Neither type of building nor vinyl wall covering was included in the final models. When type of foundation was included in the analyses (data available only for single-family houses), the associations in Table 4 remained.

## Discussion

### Measured concentrations.

The concentrations of phthalate esters are somewhat higher in our study than in some of the other studies (Table 5). This may reflect more frequent use of PVC flooring in Sweden than in other countries. Additionally, we suspect that dust samples collected by filter methods contain smaller dust particles than those obtained from vacuum cleaner bags; for semivolatile organic compounds associated with the dust via sorption processes, this would mean higher dust concentrations for filter samples compared with samples from vacuum cleaner bags. The surface from which the dust is collected can also influence the resulting chemical constituents of the dust. The highest median DEHP concentration in Table 5 is for samples collected from flooring in schools (Clausen et al. 2003). In a subsequent study, Clausen et al. (2004) presented results that indicate direct transfer of DEHP from PVC flooring to dust in contact with the PVC flooring. Finally, the method of extraction and analysis can also influence the measured concentrations.

### Associated building characteristics.

High concentrations (above median) of BBzP and DEHP in dust were associated with PVC flooring; however, BBzP was more strongly associated with PVC than was DEHP. Furthermore, BBzP was associated with self-reported water leakage, and DEHP was, to a lesser degree, associated with construction before 1960.

PVC flooring appears to be a source for both BBzP and DEHP in settled dust. The more rooms with PVC, the higher the concentration of these phthalates in dust. However, for both the phthalates, there is a “background” concentration (geometric means: DEHP, 0.5 mg/g dust; BBzP, 0.1 mg/g dust) in buildings with no PVC flooring (except for the bathroom). This is consistent with other known sources for phthalates in indoor dust.

Vinyl materials on walls were associated with a higher concentration of DEHP, but not BBzP, in dust based on the univariate analysis. However, the association disappeared in the multivariate model. This could reflect the few rooms with vinyl on walls and the fact that most of the bedrooms with vinyl on walls had PVC as flooring materials. Emission of DEHP from vinyl materials has been shown in other studies (Afshari et al. 2004; Fujii et al. 2003).

The correlation between DnBP, BBzP, and DEHP was not high, which implies that PVC materials can be plasticized with one or more of these phthalates, but that it is not routinely plasticized with a fixed ratio of these.

### Ventilation rate.

In crude analysis, there was an association between a high DEHP concentration in dust and a higher mean ventilation rate in the child’s bedroom. However, in the adjusted analysis, such an association disappeared, probably because of confounding mechanisms; for example, a higher ventilation rate is associated with an earlier construction period as well as several other building-related factors, as described elsewhere (Bornehag et al. 2005b).

### Construction period.

Buildings constructed before 1960 were found to have higher concentrations of DEHP than buildings from later periods. Such a finding could be due to a larger content of DEHP in older flooring materials (PVC), but there was no correlation between the concentration of different phthalates in dust and the age of the PVC flooring (data not shown). However, the Swedish Chemicals Inspectorate (KemI) reports that the total consumption of DEHP has decreased in Sweden over the past years (KemI 2004).

### Water leakage and change of flooring materials.

In the multiple regression analyses, water leakage during the previous 3 years was associated with an elevated concentration of BBzP in the dust. It should be stressed that the data regarding water leakage was self-reported by the parents, and that there was an 18- to 24-month interval between reports of water leakage and the exposure measurements. The association could be due to degradation of PVC floors caused by moisture/water and, in some cases, highly basic (high pH) moist concrete surfaces. On the other hand, reports of water damage may be a proxy for renovations in which old flooring materials have been replaced by new materials. Thus, there are several possible explanations regarding the association between BBzP concentration in dust and water leakage.

In this study we focused on only two indoor sources of phthalates, PVC flooring and vinyl wall covering. A typical home contains numerous other materials that are plasticized with phthalates. Examples include furniture covered with synthetic leather, vinyl raincoats, vinyl notebook covers, toys and sports equipment made of PVC, vinyl lampshades, vinyl garment bags, PVC containers, and PVC insulation on telephone, television, and computer cables. The building characteristics examined in this study cannot be proxies for these quite varied sources. However, the associations reported in this study can help to estimate, without chemical analyses, whether high or low BBzP and DEHP levels can be anticipated in a home’s dust.

## Conclusions

The main finding from this study is that the concentrations of BBzP and DEHP in dust are associated with the amount of PVC/vinyl used as flooring and wall material in the home, but that there are also many other sources of these phthalates. Although PVC flooring and vinyl on walls do not fully explain the concentration of phthalates in dust, occurrences of such materials are associated with higher concentrations of DEHP and BBzP in dust indoors. There are also associations between the concentration of BBzP in bedroom dust and water leakage in the previous 3 years, as well as higher levels of DEHP in bedroom dust and buildings constructed before 1960. The reason for the association between high BBzP concentration and self-reported water leakage is not obvious. The finding that DEHP was higher for buildings erected before 1960 could reflect higher fractional concentrations in older products or higher emission rates as products degrade.

## Figures and Tables

**Table 1:** Description of the 390 homes in the case–control study.

	No. of buildings with different characteristics (%)			
Building characteristics^a^	Single-family houses	Chain houses	Multifamily houses	Total
No. of buildings in the study	323 (82.8)	23 (5.9)	44 (11.3)	390 (100)
Flooring material in child’s bedroom				
PVC	167 (52.0)	12 (52.2)	32 (72.7)	211 (54.4)
Wood/parquet	108 (33.6)	7 (30.4)	5 (11.4)	120 (30.9)
Laminate	34 (10.6)	2 (8.7)	3 (6.8)	39 (10.1)
Linoleum	8 (2.5)	1 (4.3)	4 (9.1)	13 (3.4)
Wall-to-wall carpet	3 (0.9)	1 (4.3)	0 (0)	4 (1.0)
Other	1 (0.3)	0 (0)	0 (0)	3 (0.8)
Wall material in child’s bedroom				
Wallpaper	230 (71.2)	12 (52.2)	39 (88.6)	281 (72.0)
Painted wallpaper	41 (12.7)	5 (21.7)	4 (9.1)	50 (12.8)
Painted glass fiber	27 (8.3)	4 (17.4)	0 (0)	31 (7.9)
Vinyl	29 (9.0)	4 (17.4)	4 (9.1)	37 (9.5)
Wood	11 (3.4)	0 (0)	0 (0)	11 (2.8)
Textile	1 (0.3)	0 (0)	0 (0)	1 (0.2)
Construction period				
Before 1940	101 (31.1)	1 (4.3)	7 (15.9)	109 (27.9)
1940–1960	58 (18.0)	2 (8.7)	10 (22.7)	70 (17.9)
1961–1970	34 (10.5)	4 (17.4)	13 (29.5)	51 (13.1)
1971–1976	46 (14.2)	6 (26.1)	3 (6.8)	55 (14.1)
1977–1983	48 (14.9)	2 (8.7)	1 (2.3)	51 (13.1)
1984–1993	29 (9.0)	6 (26.1)	7 (15.9)	42 (10.8)
After 1993	7 (2.2)	2 (8.7)	3 (6.8)	12 (3.1)
Ventilation system				
Natural including kitchen fan	233 (74.4)	6 (28.6)	10 (22.7)	249 (65.9)
Mechanical exhaust	51 (16.3)	11 (52.4)	30 (68.2)	92 (24.3)
Mechanical exhaust and supply	29 (9.3)	4 (19.0)	4 (9.1)	37 (9.8)
Self-reported water leakage^b^				
Yes, during previous 3 years	68 (21.5)	8 (34.8)	7 (16.7)	83 (21.7)
No	222 (70.0)	13 (56.5)	28 (66.7)	263 (68.8)
Don’t know	27 (8.5)	2 (8.7)	7 (16.7)	36 (9.4)

a Data from inspections of the buildings in DBH phase 2 except for flooding, which was collected in the first questionnaire in DBH phase 1.

b Data from questionnaire investigation in DBH phase 1, which was collected 18 months before the exposure measurements were conducted.

**Table 2:** Concentrations (mg/g dust) for different phthalates in settled dust from 346 bedrooms.

		All samples (n = 346)				Type of flooring^a^ (median mg/g dust)		
Phthalate	Above detection limit^b^ [n (%)]	Mean	Median	Min–Max	95th percentile	No PVC (n = 157)	PVC (n = 187)	p-Value^c^
DEP	32 (9.2)	0.031	0.000	0.000–2.425	0.115	0.000	0.000	0.241
DINP	173 (50.0)	0.639	0.041	0.000–40.667	1.930	0.000	0.082	0.394
DIBP	188 (54.3)	0.097	0.045	0.000–3.810	0.311	0.042	0.050	0.120
BBzP	272 (78.6)	0.319	0.135	0.000–45.549	0.599	0.089	0.192	< 0.001
DnBP	308 (89.0)	0.226	0.150	0.000–5.446	0.568	0.133	0.159	0.138
DEHP	343 (99.1)	1.310	0.770	0.000–40.459	4.069	0.700	0.868	0.001

Abbreviations: Max, maximum; Min, minimum.

a Type of flooring in the child’s bedroom.

b Number of samples with a concentration greater than the detection limits (0.040 mg/g dust).

c Mann-Whitney U-test regarding differences in phthalate concentration between bedrooms with and without PVC as flooring material.

**Table 3:** Frequency of surface materials in the child’s bedroom (floors and walls) among cases and controls [n (%)].

Surface material	Cases	Controls
Flooring material		
PVC	118 (59.6)	97 (48.8)
Wood	47 (23.7)	76 (38.0)
Laminate	24 (12.1)	18 (9.0)
Linoleum	6 (3.0)	7 (3.5)
Wall-to-wall carpet	2 (1.0)	2 (1.0)
Other	1 (0.5)	0 (0)
Wall material		
Wallpaper	143 (72.2)	142 (70.2)
Painted wallpaper	35 (17.7)	19 (9.4)
Painted glass fiber	12 (6.0)	21 (10.4)
Vinyl	19 (9.6)	20 (9.9)
Wood	4 (2.0)	8 (3.9)
Textile	0 (0)	1 (0.5)

**Table 4:** Association between concentration of phthalates in dust (> median) and building characteristics.

		Odds ratio (95% CI)^a^	
Factor	No.	BBzP^b^	DEHP^c^
PVC as flooring			
No	138	1.0	1.0
Yes	165	3.85 (2.37–6.24)	1.85 (1.15–2.98)
Vinyl as wall material			
No	282	1.0	1.0
Yes	21	NS	NS
Type of building			
Single-family house	277	1.0	1.0
Multifamily house	26	NS	NS
Construction period			
Before 1960	144	NS	2.30 (1.17–4.52)
1960–1983	110	NS	1.09 (0.55–2.18)
After 1983	49	1.0	1.0
Ventilation rate in child’s bedroom			
1st quartile	74	NS	NS
2nd quartile	79	NS	NS
3rd quartile	80	NS	NS
4th quartile	70	1.0	1.0
Water leakage during previous 3 years			
No	227	1.0	1.0
Yes	76	1.84 (1.05–3.22)	NS

a Backward conditional logistic regression in two different models. Only significant variables included in the final model; variables with no significant contribution to the model have been eliminated (NS).

b Model 1: Dependent variable BBzP coded as 1 ≤median concentration and 2 > median concentration.

c Model 2: Dependent variable DEHP coded as 1 ≤median concentration and 2 > median concentration.

**Table 5:** Measurements of the concentration of phthalates in dust in different countries.

			DEHP (μg/g dust)		BBzP (μg/g dust)		DnBP (μg/g dust)		
Study	Country	No.	50th^a^	95th^a^	50th^a^	95th^a^	50th^a^	95th^a^	Sampling technique
Present study	Sweden	346	770	4,069	135	599	150	568	Surface dust above floor (filter)^b^
Pohner et al. 1997	Germany	272	450	2,000	—	—	—	—	“Fine dust”?
Oie et al. 1997	Norway	38	640^c^	—	110^c^	—	100^c^	—	Surface dust (filter)^d^
Butte et al. 2001	Germany	286	740	2,600	49	320	49	240	Vacuum cleaner bags
Becker et al. 2002	Germany	199	416	1,190	15	207	42	160	Vacuum cleaner bags
Clausen et al. 2003	Denmark	23	858	2,595	—	—	—	—	Floor dust (cyclone/glass bottle)
Rudel et al. 2003	USA	120	340	854^e^	45	277^e^	20	44^e^	Surface dust (filter)^d^
Kersten and Reich 2003	Germany	65	600	1,600	19	230	47	180	Vacuum cleaner bags
Fromme et al. 2004	Germany	30	703	1,540	30	218	56	130	Vacuum cleaner bags
Becker et al. 2004	Germany	252	515	1,840	—	—	—	—	Vacuum cleaner bags

a 50th, 95th: 50th and 95th percentiles.

b Multiple surfaces excluding floors.

c Mean concentration.

d Multiple surfaces including floors.

e 90% percentile.
